# Fecal microbiota transplantation from different pig breeds alters fat deposition and gut microbiota in mice

**DOI:** 10.1007/s00253-026-13823-z

**Published:** 2026-05-04

**Authors:** Fan Yang, Shihao Liu, Guoqing Liu, Liming Luo, Xvyang Lu, Weimin Lin, Jing Chen, Ruiyi Lin

**Affiliations:** Engineering Research Center for Animal Breeding and Sustainable Production, College of Animal Sciences, Fujian Agriculture and Forestry University, Fuzhou, 350002 Fujian China

**Keywords:** Gut microbiota, Fecal microbiota transplantation, Fat deposition, Putian black pigs, Duroc $\times$ Landrace $\times$ Yorkshire pigs

## Abstract

**Abstract:**

Gut microbiota plays a vital role in nutrient digestion, energy metabolism, and immune regulation in pigs. However, the core bacterial species influencing fat deposition remain poorly defined due to the complexity and diversity of the intestinal microbial ecosystem. In this study, healthy Putian Black (PT) pigs and Duroc $\times$ Landrace $\times$ Yorkshire (DLY) pigs of similar ages were used as fecal microbiota transplantation (FMT) donors, with male ICR mice as recipients. A control group (CM) received saline, while the experimental groups were gavaged fecal suspensions from PT pigs (PM) or DLY pigs (DM). Results showed that intramuscular fat content, triglyceride levels, and adipogenic gene expression (*PPARG, FABP4, LPL, ATGL*) were extremely significant higher in the PM group than in the DM group (*P*<0.01). 16 S rRNA sequencing revealed that both PM and DM groups had lower *Firmicutes* abundance but higher *Bacteroidetes* abundance compared to the CM group (*P*<0.05). Notably, the PM group exhibited higher *Firmicutes* and lower *Bacteroidetes* abundance than the DM group (*P*<0.05). Correlation analysis identified *S_uncultured_bacterium_g_Prevotella* as negatively correlated with *FASN* and *DGAT2* expression (*P*<0.01), while *Lactobacillus* species showed positive correlations with *PPARG, FASN*, and *ATGL* expression (*P*<0.05). These findings demonstrate that FMT alters gut microbiota composition and host gene expression, thereby influencing fat deposition, with *Prevotella* and *Lactobacillus* emerging as potential key genera.

**Key points:**

$\bullet$ *FMT resulted in extremely significant higher intramuscular fat content in the PM group compared to the DM group.*

$\bullet$ *Lactobacillus may be a key genus regulating fat deposition in PT pigs.*

$\bullet$ *Prevotella may be a key genus regulating fat deposition in DLY pigs.*

**Supplementary Information:**

The online version contains supplementary material available at 10.1007/s00253-026-13823-z.

## Introduction

With the rapid development of the economy, consumer demand for high-quality pork is increasing. Fat content is one of the key indicators for evaluating pork quality, as it directly affects meat flavor, tenderness, and juiciness, thereby influencing consumer preference and market price (Chernukha et al. 2023). Chinese indigenous pig breeds generally exhibit greater backfat thickness and higher intramuscular fat (IMF) content compared to commercial breeds (Zhang et al. 2022). Fat deposition in pigs is a complex physiological process regulated by multiple intrinsic and extrinsic factors, including genetic background, nutritional status, gut microbiota composition, and management practices, all of which contribute to variations in lipid synthesis and metabolism (Xiong 2025).

Adipose tissue is a loose connective tissue located beneath the skin (subcutaneous) and surrounding internal organs and tissues (Mancuso 2016). It primarily consists of triglycerides (TG) and fatty acids (FA), serving as the main energy storage component in animals. Based on its physiological function, animal adipose tissue can be classified into two major types: white adipose tissue (WAT) and brown adipose tissue (BAT) (Rosen and Spiegelman 2014). WAT mainly functions as an energy reservoir, storing excess energy and releasing it when required by the body (Park 2014), whereas BAT is primarily responsible for thermogenesis, helping maintain body temperature and prevent hypothermia (Labbe et al. 2015). According to deposition sites, adipose tissue in pigs can be categorized into subcutaneous adipose tissue (SAT), visceral adipose tissue (VAT), and IMF. VAT, located in the mesentery and omentum, is the earliest form of fat deposition in pigs (Schumacher et al. 2022). SAT, which lies beneath the skin, is mainly distributed along the back, abdomen, and flanks, forming the outer fat layer of the animal. SAT accounts for more than 70% of total body fat in pigs and is a key determinant of live pig production efficiency (Hausman 2018). Compared with muscle tissue, adipose tissue has higher energy density; thus, reducing SAT deposition can effectively improve feed conversion efficiency and enhance the economic performance of live pig production, which has long been a major objective in pig breeding programs (Wajchenberg 2000; Wajchenberg et al. 2002). IMF refers to fat deposited within muscle tissue, gradually accumulating between muscle bundles and fibers along with connective tissue development. Increasing IMF content has been shown to significantly enhance meat brightness, yellowness, and tenderness (Chartrin et al. 2006), reduce drip loss, and improve marbling score, thereby contributing to superior sensory quality (Fernandez et al. 1999).

The gut microbiota, a complex community of microorganisms including bacteria, fungi, and viruses, lives in a symbiotic relationship with the host (Wang et al. 2025). Recently, increasing research attention has been directed toward the role of the gut microbiota in regulating host lipid absorption and fat deposition, and its functional importance has been widely recognized (Kimura et al. 2013). The regulatory effects of the gut microbiota on host fat deposition are primarily mediated through mechanisms such as polysaccharide fermentation, energy metabolism, and the synthesis of short-chain fatty acids. Therefore, targeting the gut microbiota as a key regulatory factor may provide new opportunities to improve live pig production efficiency. The composition and structure of the pig gut microbiota are strongly influenced by genetic factors, and significant differences have been observed among breeds with different genetic backgrounds (Li et al. 2023; Kumar et al. 2025). Furthermore, variations in the intestinal microbial community have been demonstrated to be significantly associated with multiple growth and production traits (Liu et al. 2022; Yin et al. 2023; Yang et al. 2024; Li et al. 2024; Liu et al. 2024).

Fecal microbiota transplantation (FMT) is a technique involving the transfer of the entire gut microbial community from a healthy donor or an autologous source to the gastrointestinal tract of a recipient. This procedure rapidly and effectively reshapes or reconstructs the recipient’s intestinal microecosystem, thereby improving gut health, enhancing intestinal function, or inducing metabolic characteristics similar to those of the donor (Li et al. 2020; Holvoet et al. 2021; Chu et al. 2021). In this study, we aimed to investigate whether FMT could transfer the fat deposition traits of different pig breeds into a mouse model.

Putian Black (PT) pig is an excellent indigenous breed originating from Fujian Province, China. It is mainly distributed in Putian, Xianyou, and the northwestern region of Fuqing. The breed was developed through long-term selective breeding by local farmers, using Fuzhou “large-eared” pigs and Minnan “small-eared” pigs (Zhang et al. 2012). PT pigs exhibit a medium body size, sparse black hair, a slightly narrow chest, a concave waist, and a drooping abdomen. They are characterized by early sexual maturity, strong disease resistance, high reproductive performance, and good adaptability to coarse feed. In contrast, the Duroc $\times$ Landrace $\times$ Yorkshire (DLY) pig is a commercial hybrid widely used in intensive pig production systems. It is known for its rapid growth rate, high lean meat percentage, and good reproductive performance, but typically exhibits lower intramuscular fat (IMF) content and inferior meat quality (Shi et al. 2025). In this study, a mouse gut microbiota model derived from PT and DLY pigs was established through fecal microbiota transplantation (FMT) to investigate the effects of gut microbiota on host fat deposition. Furthermore, this study aimed to identify potential bacterial taxa involved in regulating lipid accumulation in pigs. The findings lay the foundation for further elucidating the role of gut microbiota in regulating fat deposition in pigs.

## Materials and methods

The protocols for all animal experiments were approved by the Experimental Animal Care and Use Committee of Fujian Agriculture and Forestry University (FAFU2013-0012), in accordance with the Regulations for the Administration of Affairs Concerning Experimental Animals (Ministry of Science and Technology, China, revised in July 2013).

### Animal information and experimental design

A total of forty-five 5-week-old male ICR mice (Wu’s Experimental Animal Company, Fuzhou, China) with similar body weights were randomly assigned into three groups (*n* = 15 per group): a control mouse group, a group transplanted with microbiota from DLY pigs (5 barrows and 5 gilts, 270 days old) (Yong Cheng, Fuzhou, China), and a group transplanted with microbiota from PT pigs (5 barrows and 5 gilts, 270 days old) (Putian Black Pig Breeding Conservation Farm, Putian, China). Each mouse was housed individually in a separate cage under controlled environmental conditions (temperature 26 ± 0.5 °C, 10 h light per day) with ad libitum access to food and water in a specific pathogen-free (SPF) environment. To deplete the native gut microbiota, mice were administered an antibiotic cocktail consisting of vancomycin (0.5 g/L), ampicillin (1.0 g/L), metronidazole (1.0 g/L), and neomycin (1.0 g/L) in the drinking water. The antibiotic solution was replaced weekly, and treatment continued for 2 weeks. After antibiotic pretreatment, the mice in the DM and PM groups were gavaged with fecal microbiota suspensions prepared from DLY and PT pig colonic contents, respectively, whereas the CM control group received physiological saline, using a gauge 10 gavage needle on an every-other-day schedule for 8 consecutive weeks. At the end of the 8-week period, six mice from each group were euthanized, and samples of the gastrocnemius muscle, subcutaneous fat, visceral fat, and colonic contents were collected.

### Fecal microbiota transplantation inoculum preparation and treatments

Fecal microbiota suspensions were prepared from DLY and PT pig fecal samples. Frozen fecal samples were thawed in a 37 °C water bath for 10 min. The samples were weighed and transferred to 50 mL centrifuge tubes, and sterile 5% phosphate-buffered saline (PBS) was added at a ratio of 1.5 mL per 100 mg of feces. The mixture was vortexed until no visible large particles remained. The resulting suspension was sequentially filtered through sterile 200-mesh, 400-mesh, and 800-mesh sieves to remove large particles, undigested feed, and other coarse debris, followed by vortexing for 5 min to resuspend the filtrate. The suspension was then centrifuged at 600 $\times$ g for 5 min to remove insoluble material, yielding the fecal microbiota suspension. Transfer 1 $\mu$L of the fecal microbiota suspension into a cuvette and measure the optical density (OD) 600 value using an ultraviolet spectrophotometer. Based on the result, adjust the suspension to an OD 600 value of 1 ± 0.05, at which point the bacterial concentration is approximately 10^8^–10^9^ CFU/mL (colony forming units/ml). Aliquot the suspension into 1.5 mL centrifuge tubes (0.9 mL per tube), add 0.1 mL of sterile glycerol, seal the tubes with sealing film, and store them in a –80 °C ultra-low temperature freezer until use.

One day prior to transplantation, No. 10 gavage needles and 1 mL syringes were sterilized by autoclaving. Before each gavage, frozen fecal microbiota suspensions were thawed in a pre-warmed 37 °C water bath. Using a 1 mL sterile syringe, the suspension was drawn and connected to the No. 10 gavage needle. The body weight of the mice was measured to calculate the gavage volume at a ratio of 10 $\mu$L per gram. This gavage procedure was performed every other day for a duration of 8 weeks. At the end of the 8-week period, following the final gavage, the mice were fasted for 12 h before colonic contents were collected.

### Growth performance

Body weight was recorded for all mice at 7-day intervals, from the initiation of the antibiotic treatment in the drinking water until the conclusion of the fecal microbiota transplantation (FMT) period. Body weight changes were used as an indicator of fat accumulation in the mice. The weight gain of the DM and PM groups was compared with that of the CM group over the entire experimental period to evaluate the effects of microbiota from different pig sources on host fat accumulation.

### Determination of intramuscular fat and triglyceride content

At the end of the 8-week fecal microbiota transplantation period, six mice were randomly selected from each group (CM, DM, and PM) for sample collection. Gastrocnemius muscles were harvested and processed into frozen sections. Intramuscular fat was visualized by Oil Red O staining, and lipid droplet content was quantified using ImageJ (Schneider et al. 2012) to compare relative intramuscular fat levels among the three groups. Triglyceride (TG) content in subcutaneous adipose tissue, visceral adipose tissue, and intramuscular fat was measured using a commercial TG assay kit (Solarbio, Beijing, China) according to the manufacturer’s instructions.

**Table 1:** Gene primer sequences

Genes	Forward primer	Reverse primer	Accession number
*GAPDH*	GTTCCAGTATGATTCCACCCAC	TTCACGCCCATCACAAACAT	NM_001206359
*PPARG*	CCAGGACTACCAAAGTGCCA	TTTATCCCCACAGACACGGC	NM_214379
*FASN*	CCTTCGATGCCGAAGGGAC	TTGGAACCGTCTGTGTTCGT	NM_001099930
*FABP4*	CATGAAAGAAGTGGGAGTGGGC	CTGGCCCAATTTGAAGGCAAT	NM_001002817
*LPL*	TCAGAGTGAAGGCAGGAGAGA	TTTGCTCAGTTTCAGCCAGA	NM_214286
*DGAT2*	GTGGWTCCTGTCTTTCCTCG	CCTCCGGCCACCTTTCTTG	NM_001160080
*ATGL*	GCTCAATGACGCCCTGCT	GCAAGCGGACGGTGAAGG	NM_001098605
*LIPE*	CACTGACTGCTGACCCCAAG	GCTCCTCACTGTCCTGTCCT	NM_214315

*Source*: Gene primer sequences for various genes

### Total RNA extraction, reverse transcription reaction, and real-time quantitative PCR

At the end of the gavage period, six mice were randomly selected from each group (CM, DM, and PM). Subcutaneous adipose tissue, visceral adipose tissue, and intramuscular fat were collected separately for total RNA extraction using the chloroform method. The purity and concentration of RNA were determined using a UV spectrophotometer, and RNA samples were adjusted to a concentration of 300 ng/$\upmu$L. The RNA samples were reverse-transcribed into cDNA using a commercial reverse transcription kit (Accuri Bio, Shanghai, China) following the manufacturer’s instructions. RT-qPCR was performed to assess the expression of fat deposition-related marker genes, including *PPARG, FASN, FABP4, LPL, DGAT2, ATGL*, and *LIPE*. Relative mRNA expression levels were calculated using the $2 ^{-\Delta \Delta Ct}$ method, with *GAPDH* serving as the internal reference gene. Primers for the target genes were designed based on pig mRNA sequences obtained from the GenBank database using NCBI tools, and synthesized by Sangon Biotech (Shanghai, China). Primer sequences are listed in (Table 1).

### Microbiota DNA extraction and 16 S rRNA sequencing

Total DNA of the gut microbiota was extracted from the intestinal contents of mice. The quality and concentration of the extracted DNA were assessed using 1.0% agarose gel electrophoresis and a NanoDrop 2000 spectrophotometer (Scientific, Waltham, MA, USA). Qualified samples were stored at –80 °C for subsequent analysis. The hypervariable V3-V4 region of the bacterial 16 S rRNA gene was amplified with primer pairs 338F (5’-ACTCCTACGGGAGGCAGCAG-3’) and 806R (5’-GGACTACHVGGGTWTCTAAT-3’) using a T100 Thermal Cycler PCR system (Bio-Read, Hercules, CA, USA).

The PCR reaction mixture (20 $\mu$L final volume) contained 4 $\mu$L of 5$\times$ Fast Pfu buffer, 2 $\mu$L of 2.5 mM dNTPs, 0.8 $\mu$L of each primer (5 $\mu$M), 0.4 $\mu$L of Fast Pfu polymerase, 10 ng of template DNA, and ddH$_2$O to volume. The PCR cycling protocol was as follows: initial denaturation at 95 °C for 3 min; 27 cycles of denaturation at 95 °C for 30 s, annealing at 55 °C for 30 s, and extension at 72 °C for 45 s; followed by a final extension at 72 °C for 10 min, with a final hold at 4 °C.

The resulting amplicons were purified, pooled in equimolar ratios, and subjected to paired-end sequencing on an Illumina NextSeq 2000 platform (Illumina, San Diego, CA, USA) by Majorbio Bio-Pharm Technology Co., Ltd. (Shanghai, China), according to the standard protocols. The raw sequencing reads have been deposited in the NCBI Sequence Read Archive database under the accession number PRJNA1210781.

### Bioinformatics analysis

Following demultiplexing of the raw sequencing data, quality control filtering was performed using fastp (v0.19.6) (Magoc and Salzberg 2011), and paired-end reads were merged using FLASH (v1.2.11) (Chen et al. 2018). These high-quality merged sequences were then subjected to denoising with the DADA2 plugin within the QIIME2 (v2020.2) (Wang et al. 2007) pipeline, following the recommended parameters, which infers amplicon sequence variants (ASVs) at single-nucleotide resolution. The metagenomic functions were predicted from the representative ASV sequences using PICRUSt2 (Douglas et al. 2020). The ASV representative sequences were integrated into a reference phylogenetic tree using EPA-NG and Gappa, and 16 S rRNA gene copy numbers were normalized using Castor. All aforementioned bioinformatic analyses were conducted on the Majorbio Cloud Platform (https://cloud.majorbio.com).

Based on the ASV table, rarefaction curves and alpha diversity indices, including the observed ASVs and the Shannon index, were calculated with Mothur (v1.30.1) (Schloss et al. 2009). The similarity of microbial communities across different samples was assessed by Principal Coordinate Analysis (PCoA) based on the Bray-Curtis dissimilarity metric using the Vegan (v2.5-3) (Dixon 2003) package. Permutational multivariate analysis of variance also implemented in Vegan, was applied to determine the proportion of variation explained by the treatment groups and its statistical significance. Additionally, Linear Discriminant Analysis Effect Size (LEfSe) (Segata et al. 2011) was employed to identify taxa (from phylum to genus) that were significantly abundant in different groups, using a Linear Discriminant Analysis (LDA) score threshold of > 2 and a statistical significance of *P*<0.05.

**Fig. 1:**
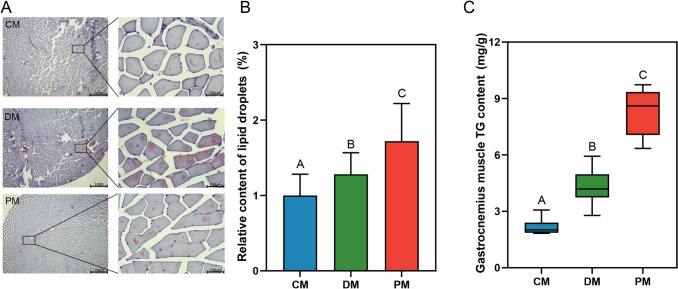
Effects of fecal microbiota transplantation (FMT) on fat deposition in mice. **A** Oil Red O staining of gastrocnemius muscle sections from the three groups of mice. **B** Relative lipid droplet content in gastrocnemius muscle. **C** Relative triglyceride content in gastrocnemius muscle. Data are presented as mean ± SEM (*n* = 6). Different lowercase letters indicate a significant difference at *P*<0.05, different uppercase letters indicate an extremely significant difference at *P*<0.01

### Statistical analysis

All data were analyzed using SPSS version 23.0 (IBM, Armonk, NY, USA) and R software (R Core Team 2021). Results were presented as mean ± standard error of the mean (SEM) and were visualized using GraphPad Prism 10 (GraphPad Software, San Diego, CA, USA). Statistical significance was evaluated using one-way analysis of variance (ANOVA). Adjusted *P*-values < 0.05 were considered statistically significant.

## Results

**Fig. 2:**
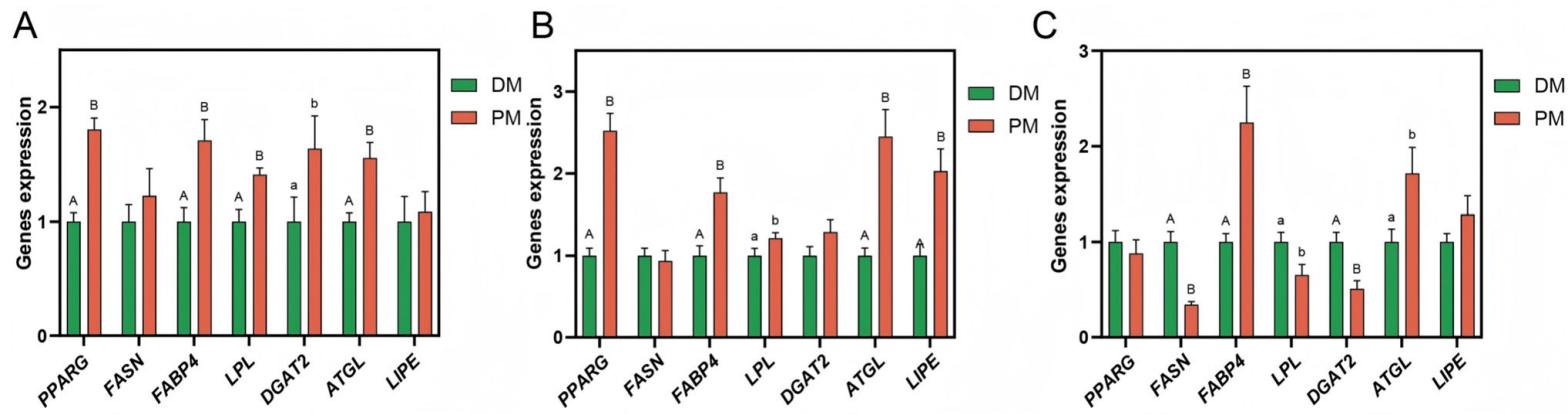
Expression levels of fat deposition–related marker genes in adipose tissues of DM and PM mice detected by RT-qPCR. **A** Intramuscular fat (IMF). **B** Visceral adipose tissue (VAT). **C** Subcutaneous adipose tissue (SAT). Data are presented as mean ± SEM (*n* = 6). Different lowercase letters indicate a significant difference at *P*<0.05, different uppercase letters indicate a extremely significant difference at *P*<0.01

**Fig. 3:**
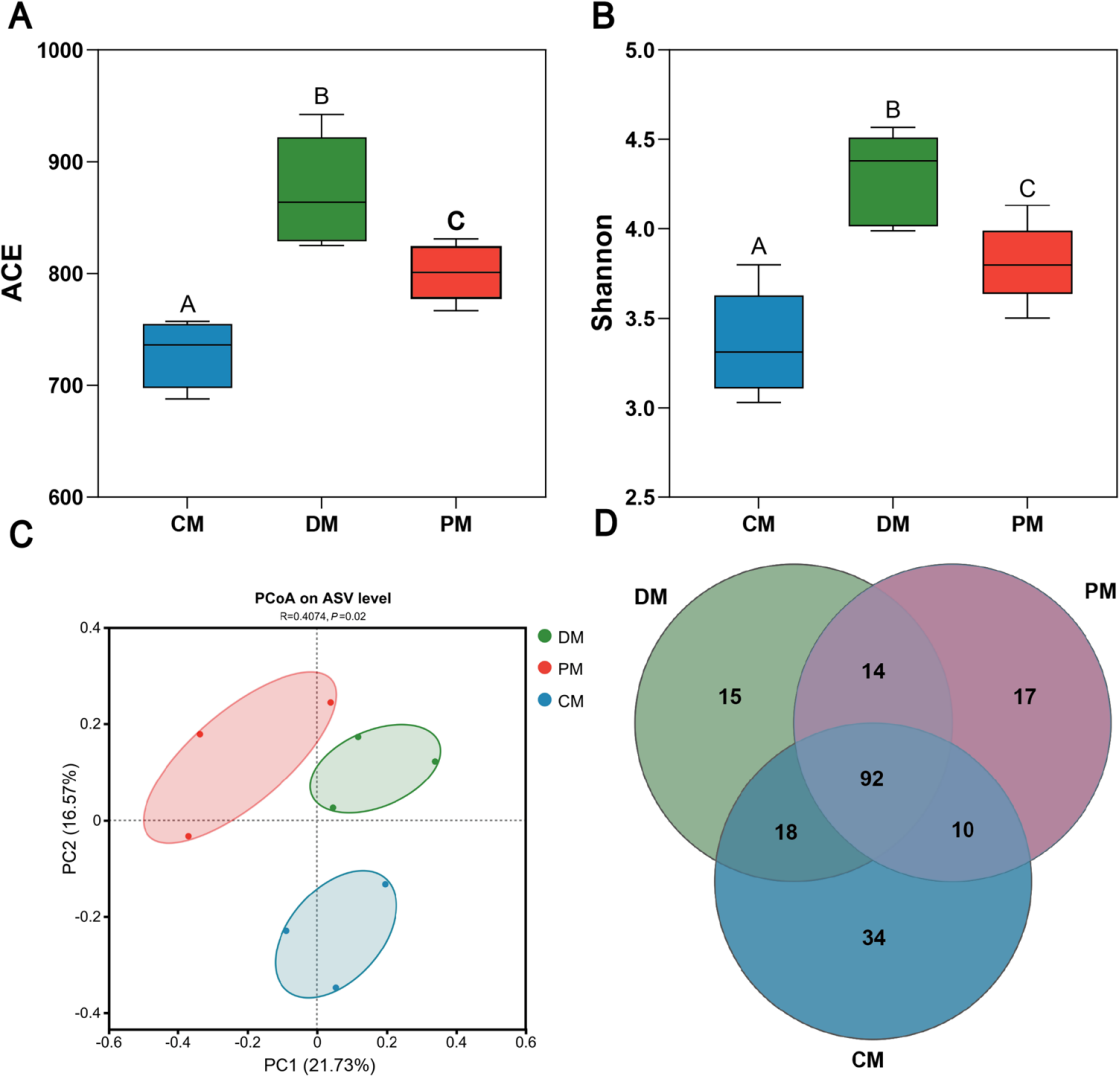
Gut microbial diversity in mice. **A** ACE index. **B** Shannon index. **C** Principal component analysis (PCA) plot. **D** Venn diagram at the genus level. Note: *n* = 3. Different superscript letters indicate extremely significant differences; capital letters denote *P*<0.01

### Effects of fecal microbiota transplantation on fat deposition in mice

To investigate differences in fat deposition among the three groups of mice, body weight changes were recorded throughout the experimental period. During the antibiotic cocktail treatment from 5 to 7 weeks of age, body weight decreased significantly in all groups, indicating effective depletion of the native gut microbiota. Throughout the subsequent gavage period (7–15 weeks of age), both the DM and PM groups demonstrated higher body weight gain compared to the CM group. From week 10 onward, the differences in body weight among the three groups became extremely significant (*P*<0.01), suggesting that FMT promoted growth and development in the recipient mice (Supplemental Fig. S1).

Oil Red O staining was performed on the gastrocnemius muscle from the three groups of mice (Fig. 1A) to assess relative lipid droplet content and triglyceride (TG) levels. The relative lipid droplet content in the gastrocnemius muscle was significantly higher in the PM group (1.72% ± 0.46%) than in the DM group (1.28% ± 0.27%) (*P*<0.05), which in turn was significantly higher than that in the CM group (1.00% ± 0.27%) (*P*<0.05) (Fig. 1B). The TG content in the gastrocnemius muscle was extremely significant higher in the PM group (8.30 ± 1.20 mg/g) compared to the DM group (4.29 ± 0.93 mg/g) (*P*<0.01), and the level in the DM group was extremely significant higher than that in the CM group (2.16 ± 0.42 mg/g) (*P*<0.01) (Fig. 1C). The results demonstrated that the FMT mice had elevated levels of intramuscular fat deposition compared to the control group, with the PM group showing a higher level than the DM group.

**Fig. 4:**
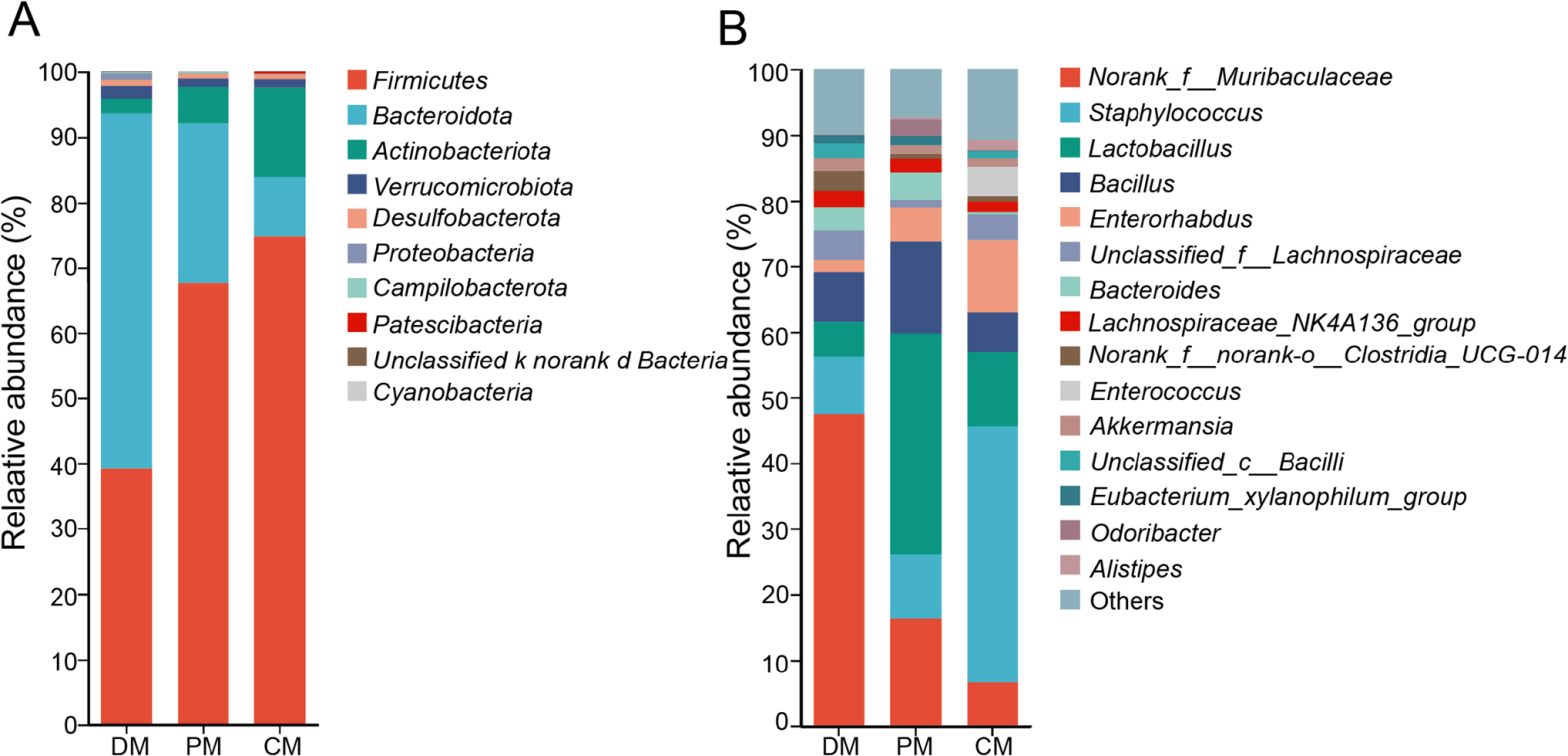
Composition of gut microbiota in mice. **A** Compositional structure of the gut microbiota at the phylum level. **B** Compositional structure of the gut microbiota at the genus level

### Effects of fecal microbiota transplantation on expression of fat deposition-related genes

The mRNA expression levels of genes related to fat deposition (*PRARG, FASN, FABP4, LPL, DGAT2, ATGL, LIPE*) were measured. In IMF, the expression levels of *PPARG, FASN, FABP4, LPL*, and *ATGL* in the PM group were extremely significant higher than those in the DM group (*P*<0.01). The expression level of *DGAT2* was significantly higher in the PM group (*P*<0.05) (Fig. 2A). In VAT, the expression levels of *PPARG, FABP4, ATGL*, and *LIPE* in the PM group were extremely significant higher than those in the DM group (*P*<0.01), and the *LPL* expression level was significantly higher (*P*<0.05) (Fig. 2B). In SAT, the expression levels of *FASN* and *DGAT2* in the PM group were extremely significant lower than those in the DM group (*P*<0.01), and the* LPL* expression level was significantly lower (*P*<0.05). In contrast, the expression level of *FABP4* was extremely significant higher (*P*<0.01), and the *ATGL* expression level was significantly higher (*P*<0.05) in the PM group (Fig. 2C).

### Gut microbiota diversity in FMT mice

To explore the potential mechanisms by which FMT influences fat deposition in mice, 16 S rRNA gene sequencing was performed to compare gut microbial diversity and composition among the PM, DM, and CM groups. Alpha and Beta diversity analyses indicated that ACE and Shannon indices differed significantly among the three groups (*P*<0.01) (Fig. 3A, B). Principal coordinates analysis (PCoA) further demonstrated distinct clustering of individual microbial communities within each group, with significant differences inter-group differences (*P*<0.05) (Fig. 3C). In addition, Venn diagram analysis indicated that the DM, PM, and CM groups harbored 15, 17, and 34 unique genera, respectively (Fig. 3D). The above results collectively indicate that there are distinct differences in the composition and structure of the gut microbiota among the PM, DM, and CM groups.

### Gut microbiota composition in FMT mice

To identify core bacterial species potentially influencing fat deposition in mice, an in-depth analysis of the 16 S rRNA sequencing data was conducted. From a taxonomic perspective, a total of 11 phyla and 200 genera were identified in the intestinal contents among the three groups. At the phylum level, compared to the CM group, the DM group showed significantly lower relative abundances of *Firmicutes* (39.19%) and *Actinobacteriota* (2.22%) (*P*<0.05), but extremely significant higher abundances of *Bacteroidota* (54.44%) and *Proteobacteria* (1.14%) (*P*<0.01). The PM group had significantly higher abundances of *Bacteroidota* (24.38%) and *Campilobacterota* (0.25%), and a significantly lower abundance of *Actinobacteriota* (5.61%) than the CM group (*P*<0.05). Furthermore, compared to the DM group, the PM group exhibited significantly higher relative abundances of *Firmicutes* (67.65%) and *Actinobacteriota* (5.61%), and significantly lower abundances of *Bacteroidota* (24.38%) and *Proteobacteria* (0.1%) (*P*<0.05) (Fig. 4A, Supplemental Table S1).

At the genus level, the DM group had significantly higher relative abundances of *Muribaculaceae* (47.39%), *Bacteroides* (3.52%), and *Prevotella* (0.95%) (*P*<0.05), but an extremely significant lower abundance of *Enterorhabdus* (1.84%) (*P*<0.01) compared to the CM group. The PM group showed significantly higher relative abundances of *Muribaculaceae* (17.98%), *Lactobacillus* (33.68%), and *Bacteroides* (4.51%), and a significantly lower abundance of *Enterorhabdus* (5.11%) than the CM group (*P*<0.05). Additionally, the PM group demonstrated significantly higher relative abundances of *Lactobacillus* (33.68%) and *Enterorhabdus* (5.11%), and significantly lower abundances of *Muribaculaceae* (17.98%) and *Prevotella* (0.28%) compared to the DM group (*P*<0.05) (Fig. 4B, Supplemental Table S2).

**Fig. 5:**
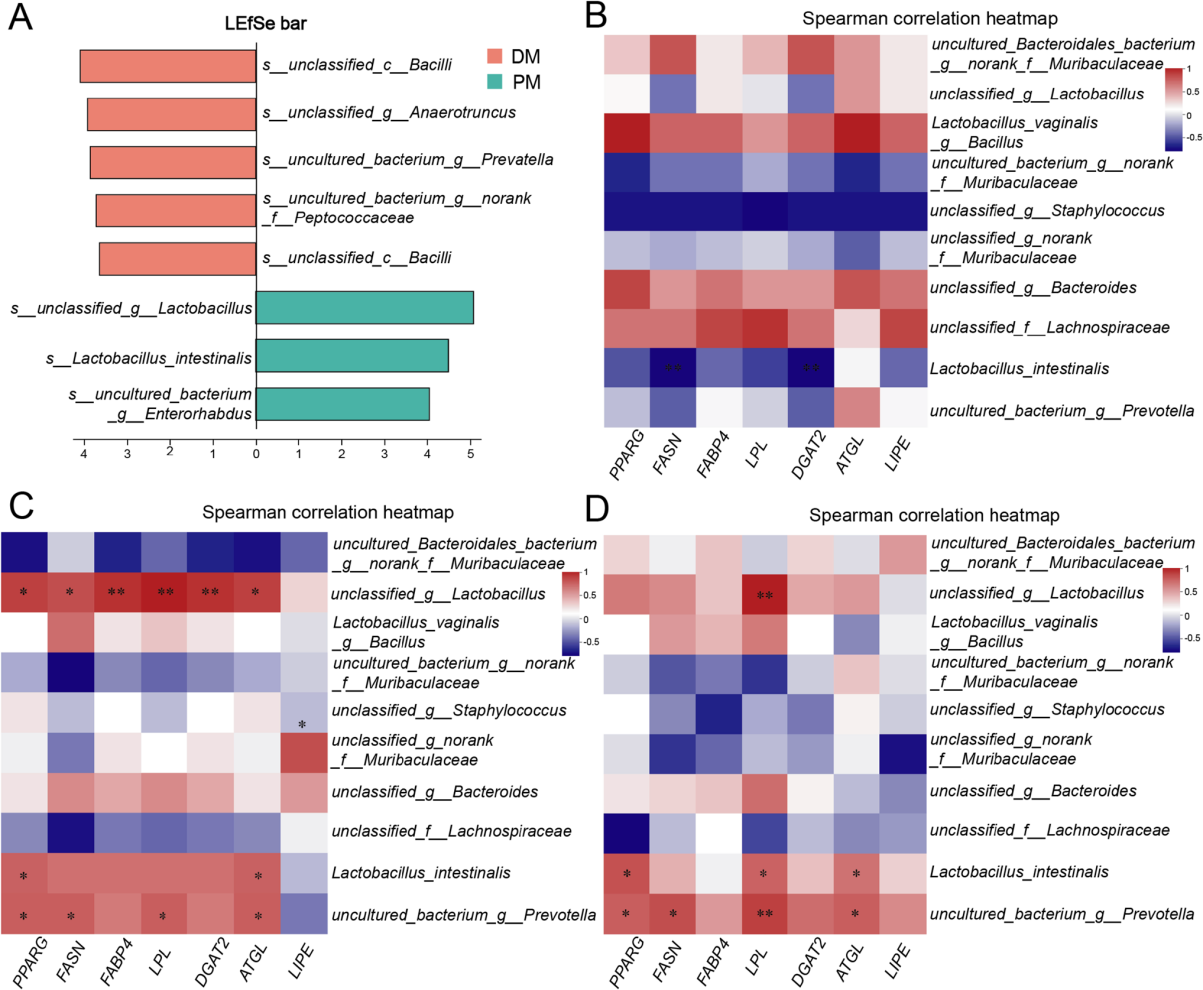
Identification and analysis of core differential bacterial species. **A** Histogram of LDA scores from LEfSe analysis, showing the core microbial taxa that were significantly enriched in either the DM or PM groups. **B–D** Heatmaps depicting the correlation between the core differential bacterial species and the expression levels of fat deposition-related marker genes in **B** IMF, **C** SAT, and **D** VAT. Data are from *n* = 3 biologically independent samples. *P<0.05, **P<0.01

### Correlation analysis between core differential bacterial species and fat-related marker genes

Core bacterial species in the DM and PM groups were identified through LEfSe analysis. It was found that *S_unclassified_c_Bacilli*, *S_unclassified_g_Anaerotruncus*, *S_uncultured_* *bacterium_g_Prevotella*, *S_uncultured_bacterium_g_norank_f_Peptococcaceae*, and *S_unclassified_c_Clostridia* were the core species in the DM group; whereas *S_unclassified_g_Lactobacillus, Lactobacillus intestinalis,* and *S_uncultured_bacterium_g_Enterorhabdus* were identified as the core species in the PM group (Fig. 5A). Furthermore, comparison with the core microbiota previously detected in DLY and PT pigs by our laboratory revealed that *S_uncultured_bacterium* *_g_Prevotella* was a shared species between DLY pigs and the DM group, and *S_unclassified_g_Lactobacillus* and *L. intestinalis* were shared between PT pigs and the PM group (the gut microbiota sequencing data of the donor pigs are presented in Pan et al. (2026)). The sequencing data have been deposited in the NCBI database under the accession number PRJNA1210778).

To investigate the impact of the identified core differential bacterial species on fat deposition in mice, a correlation analysis was conducted between these core species and the expression levels of fat marker genes in different adipose tissues. In SAT, *L. intestinalis* showed extremely significant negative correlation with the expression levels of *FASN* and *DGAT2* (*P*<0.01). *S_unclassified_g_Lactobacillus* and *Prevotella* did not show significant correlations with any fat marker genes (*P*<0.05), although they indicated a trend toward negative correlation with *FASN* and *DGAT2* expression (Fig. 5B).

In VAT, *S_unclassified_g_Lactobacillus* was extremely significant positively correlated with the expression levels of *FABP4*, *LPL*, and *DGAT2* (*P*<0.01), and significantly positively correlated with *PPARG, FASN,* and *ATGL* expression (*P*<0.05). *L. intestinalis* was significantly positively correlated with the expression of *PPARG* and *ATGL* (*P*<0.05). *S_uncultured_bacterium_g_Prevotella* was significantly positively correlated with the expression levels of *PPARG, FASN*, *LPL*, and *ATGL* (*P*<0.05) (Fig. 5C).

In IMF, *S_unclassified_g_Lactobacillus* was extremely significant positively correlated with *LPL* expression (*P*<0.01). *L. intestinalis* was significantly positively correlated with the expression levels of *PPARG, LPL,* and *ATGL* (*P*<0.05). *S_uncultured_bacterium_g_Prevotella* was extremely significant positively correlated with *LPL* expression (*P*<0.01) and significantly positively correlated with the expression of *PPARG, FASN,* and *ATGL* (*P*<0.05) (Fig. 5D). These results indicate that *S_uncultured_bacterium_g_Prevotella, S_unclassified_g_Lactobacillus,* and *L. intestinalis* can regulate fat deposition in different body sites by influencing the expression of fat marker genes.

## Discussion

The gut microbiota of pigs is closely associated with their production performance. Fecal microbiota transplantation (FMT), which involves transferring porcine fecal microbiota into germ-free or antibiotic-treated mice, is a widely used approach to investigate the functional effects of pig gut microbiota on host productivity. This technique enables the establishment of a direct causal link between the gut microbiota and host production traits (Tremaroli and Bäckhed 2012; Duca et al. 2014). Previous evidence suggests that gut microbes may directly regulate host fat deposition (Xie et al. 2022b, a). To identify specific gut microorganisms influencing porcine fat deposition while controlling for host genetic effects, mouse models colonized with fecal microbiota derived from different pig breeds were established. By comparing fat deposition levels and gut microbial composition between mice colonized with distinct porcine microbiota and control mice, core bacterial species potentially responsible for regulating host fat accumulation were identified.

The key to constructing an effective mouse model lies in whether the gut microbiota of the recipient mice can successfully establish a community structure similar to that of the donor pigs, and whether the phenotypic differences associated with the target traits can be recapitulated in the recipient mice. In this study, the gut microbial composition and structure of FMT-treated mice were significantly different from those of the control mice (*P*<0.05). Moreover, significant differences in microbial community composition and structure were also observed between mice colonized with microbiota from different pig donors (*P*<0.05). The composition and structure of the gut microbiota in recipient mice showed a certain similarity to that of the donor pigs, which was consistent with previous findings that FMT can successfully transplant the gut microbiota characteristics of donor pigs into recipient mice (Zhang et al. 2020; Su et al. 2021; Hinchliffe et al. 2022). However, these studies also emphasized that discrepancies may still exist between donor and recipient microbiota, particularly in the relative abundance of specific bacterial taxa. Similar patterns were observed in our experiment, where certain bacterial phyla and dominant genera differed between donor pigs and recipient mice. These discrepancies may be attributed to species-specific colonization preferences; for example, *Spirochaetota* and *Verrucomicrobiota* are predominantly established in the gastrointestinal tracts of ruminants and pigs, whereas their colonization is relatively limited in mice (Paster and Canale-Parola 1982; Niu et al. 2015).

By comparing gene expression between mice colonized with gut microbiota from fat-type and lean-type pigs, it was observed that the expression levels of *LPL* and *FASN* in muscle were significantly higher in mice receiving microbiota from fat-type pigs than in those receiving microbiota from lean-type pigs. Similarly, Xie et al. (2022b) reported that the expression levels of *DGAT2* and *FASN* in the skeletal muscle of mice gavaged with mixed fecal microbiota were significantly higher than those of control mice (*P*<0.05). In this study, the IMF content of FMT recipient mice was significantly higher than that of control mice (*P*<0.01), and the IMF content of the PM group was extremely significant higher than that of the DM group (*P*<0.01). Furthermore, the expression levels of multiple adipogenic marker genes in SAT, VAT, and IMF were significantly higher in the PM group than in the DM group (*P*<0.05). The expression trends of these genes across different tissues were also consistent with those observed between DLY and PT pigs, suggesting that the gut microbiota of DLY and PT pigs may regulate host fat deposition by influencing the expression of adipogenic marker genes. The PM group displayed higher expression of *LPL* in IMF, but lower expression in SAT, compared to the DM group, while the study by Lian et al. (2007) indicates a significant positive correlation with IMF deposition levels, and the expression patterns of *DGAT2* and *FASN* suggest that this may explain the reason.

The gastrointestinal tract of animals harbors tens of thousands of microorganisms that play essential roles in host physiology, including immune regulation and metabolic processes, thereby exerting profound effects on growth and development (Zakria et al. 2025; Kadhim and Hasan 2025). In the present study, *Prevotella*, *L. intestinalis*, and *S_unclassified_g_Lactobacillus* derived from porcine gut microbiota were found to be significantly correlated with the expression of adipogenic marker genes. *Prevotella* is a key carbohydrate-degrading genus capable of breaking down complex polysaccharides and producing short-chain fatty acids, which serve as important energy sources for the host (White et al. 2014). Several studies have reported significant differences in the abundance of *Prevotella* between fat-type and lean-type pig breeds (Yan et al. 2025; Zhou et al. 2025). Chen et al. (2021) demonstrated that *Prevotella* in the porcine gut microbiota can activate host chronic inflammatory responses through the TLR4 and mTOR signaling pathways via microbial metabolites, thereby markedly increasing host fat deposition. Similarly, Zhang et al. (2025) identified *Prevotella* as a potential biomarker associated with intramuscular fat (IMF) content in Jinhua pigs. *Lactobacillus* can produce organic acids, such as lactic acid, to lower intestinal pH, inhibit the growth of pathogenic bacteria, and maintain intestinal microbial homeostasis, thereby preventing diarrhea, constipation, and other dysbiosis-related diseases (Kadhim and Hasan 2025). Moreover, *Lactobacillus* can degrade specific dietary components, release nutrients more readily absorbed by the host, and stimulate intestinal epithelial cells to secrete digestive enzymes, enhancing nutrient digestion and absorption. Previous studies have shown that *Lactobacillus* plays an important role in regulating fat deposition in broilers. For instance, *Lactobacillus johnsonii* BS15 was reported to improve lipid metabolism, intestinal development, and gut microbial composition, thereby promoting growth performance while reducing fat accumulation (Wang et al. 2017). Similarly, Shang et al. (2022) identified *Lactobacillus* and *Solobacterium* as key bacterial groups involved in fat deposition in Tibetan pigs. These findings are consistent with the results of our study.

## Conclusions

The gut microbiota derived from different pig sources influenced fat deposition in mice, with *S_uncultured_bacterium_* *g_Prevotella*, *L. intestinalis*, and *S_unclassified_g_Lactobacillus* identified as potential core species modulating fat deposition capacity in pigs. This study provides new insights into how the gut microbiota regulates adipose deposition.

## Supplementary Information

Below is the link to the electronic supplementary material.

Supplementary file 1 (pdf 149 KB)

## Data Availability

Publicly available in a repository: The 16 S rRNA raw sequencing reads have been deposited in the NCBI Sequence Read Archive database under the accession number PRJNA1210781.
