# Supplementary material for: Fecal microbiota transplantation from different pig breeds alters fat deposition and gut microbiota in mice
Source: Appl Microbiol Biotechnol. 2026 May 4;110(1):186. doi: 10.1007/s00253-026-13823-z (PMC13284016; doi:10.1007/s00253-026-13823-z)
Supplement: Supplementary file 1 — (pdf 149 KB) [file 253_2026_13823_MOESM1_ESM.pdf]

Applied Microbiology and Biotechnology

Supplementary material

Fecal Microbiota Transplantation from Different Pig Breeds Alters Fat Deposition and Gut Microbiota in Mice

Fan Yang, Shihao Liu, Guoqing Liu, Liming Luo, Xvyang Lu, Weimin Lin, Jing Chen, Corresponding author:Ruiyi Lin*

Engineering Research Center for Animal Breeding and Sustainable Production, College of Animal Sciences, Fujian Agriculture and Forestry University, Fuzhou, 350002, Fujian, China.

Corresponding author email:linruiyi@fafu.edu.cn

Figure

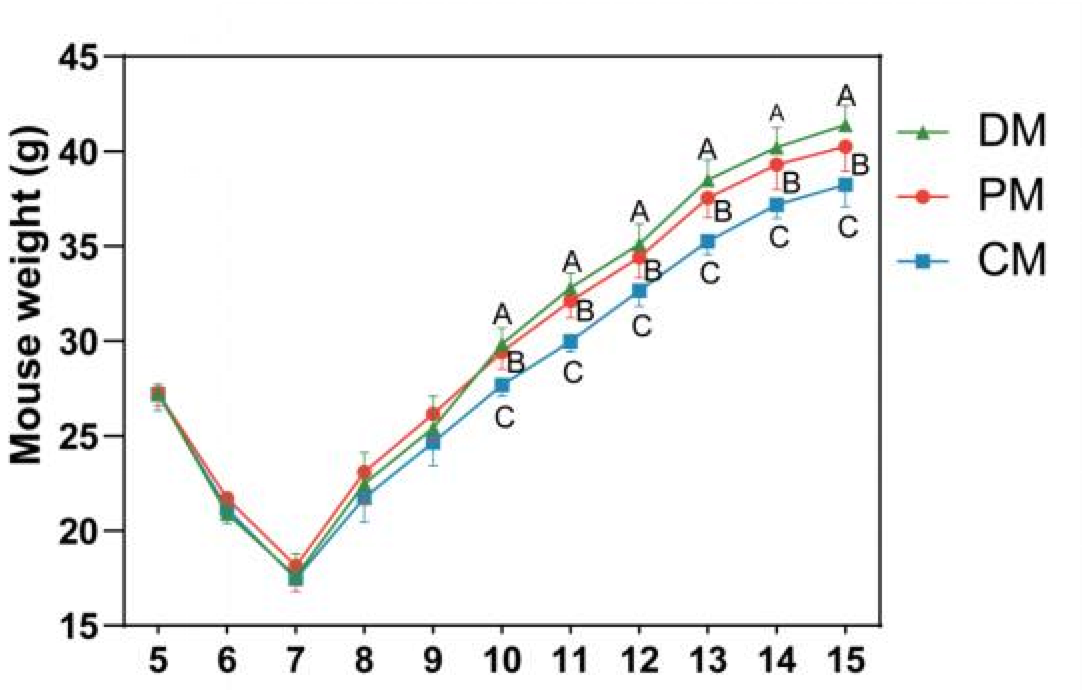

Fig. S1: Body weight changes of mice during antibiotic treatment (weeks 5–7) and gavage period (weeks 7–15). Note: n = 15; different superscript letters indicate significant differences, and uppercase letters denote $P < 0.01$.

Table

Table S1: Microbiota composition at the phylum level

Phylum	CM	DM	PM
Firmicutes	74.79±2.84%^{A}	39.19±7.59%^{Ba}	67.65±9.39%^{b}
Bacteroidota	9.15±1.41%^{Aa}	54.44±8.06%^{Ba}	24.38±8.61%^{b}
Actinobacteriota	13.6±2.92%^{a}	2.22±0.69%^{b}	5.61±1.52%^{c}
Proteobacteria	0.06±0.04%^{a}	1.14±0.59%^{b}	0.1±0.02%^{a}
Campilobacterota	0.06±0.08%^{a}	0.2±0.26%	0.25±0.07%^{b}

Source: Core differential bacterial phyla in the gut microbiota of CM, DM, and PM groups. Note: n = 3; different superscript letters indicate significant differences, with lowercase letters denoting $P < 0.05$ and uppercase letters denoting $P < 0.01$. Data are presented as mean ± SEM.

Table S2. Microbiota composition at the genus level

Genus	CM	DM	PM
norank_f_Muribaculaceae	6.58±2.49%^{A}	47.39±8.32%^{B}	17.98±4.1%^{C}
Lactobacillus	11.41±5.73%^{A}	5.28±4.06%^{AB}	33.68±5.32%^{C}
Enterorhabdus	10.97±3.64%^{A}	1.84±0.42%^{Ba}	5.11±1.42%^{b}
Bacteroides	0.48±0.43%^{a}	3.52±0.23%^{b}	4.51±1.93%^{b}
Prevotella	0.2±0.18%^{a}	0.95±0.27%^{b}	0.28±0.29%^{a}

Source: Core differential bacterial genera in the gut microbiota of CM, DM, and PM groups. Note: n = 3; different superscript letters indicate significant differences, with lowercase letters denoting $P < 0.05$ and uppercase letters denoting $P < 0.01$. Data are presented as mean ± SEM.
